# Case Report: Giant undifferentiated pleomorphic sarcoma of the breast combined with contralateral high-grade intraductal carcinoma

**DOI:** 10.3389/fonc.2026.1633750

**Published:** 2026-04-10

**Authors:** Zhikun Zhao, Lei Cao, Shengju Xu, Fan Yang, Shang Gao, Chuanxin Ren, Xingchao Xu, Xiangqi Li

**Affiliations:** 1 Department of General Surgery, Second Affiliated Hospital of Shandong First Medical University, Tai'an, Shandong, China; 2 Department of Breast Surgery, The Affiliated Taian City Central Hospital of Qingdao University, Tai'an, Shandong, China; 3 Department of Obstetrics and Gynecology, The Second Affiliated Hospital of Shandong First Medical University, Tai'an, Shandong, China; 4 Department of Pathology, The Second Affiliated Hospital of Shandong First Medical University, Tai'an, Shandong, China; 5 Department of Breast Surgery, The Second Affiliated Hospital of Shandong First Medical University, Shandong, Tai'an, China

**Keywords:** breast cancer, case report, high-grade intraductal carcinoma, multidisciplinary treatment, undifferentiated pleomorphic sarcoma

## Abstract

This article reports an exceptionally rare case of a 43-year-old female presenting with concurrent giant pleomorphic undifferentiated sarcoma and high-grade intraductal carcinoma of the contralateral breast. The tumor, remarkably large and comparable in size to a basketball, presented significant diagnostic and therapeutic challenges. Following a multidisciplinary consultation, the patient underwent a comprehensive treatment regimen, which included left mastectomy, modified radical mastectomy, vacuum-assisted closure (VSD) negative pressure drainage, radiotherapy, and endocrine therapy for the high-grade intraductal carcinoma. Post-treatment follow-up over a period of 2 to 5 years revealed a satisfactory recovery with no evidence of recurrence or metastasis. This case underscores the critical role of a multidisciplinary approach in managing rare and complex breast malignancies and highlights the potential for favorable outcomes even in cases involving exceptionally large tumors.

## Background

Breast sarcoma is a relatively rare type of malignant breast tumor, primarily originating from the mesenchymal tissues of the breast, including fibrous, adipose, and muscular tissues (1). It is distinctly different from breast cancer, which originates from epithelial cells, in terms of histological origin. Breast sarcoma can be classified into various types based on histology, with the most common being fibrosarcoma, liposarcoma, rhabdomyosarcoma, and angiosarcoma. Breast sarcoma constitutes a relatively low proportion of malignant breast tumors, approximately 1% (2).

The exact etiology of breast sarcoma remains poorly understood but is generally thought to be related to several factors (3). Mesenchymal cells in breast tissue may undergo gene mutations or chromosomal abnormalities due to certain carcinogenic factors, leading to abnormal cell proliferation and differentiation. Risk factors include a history of chest radiotherapy, long-term exposure to chemicals such as polycyclic aromatic hydrocarbons and aromatic amines (4).

Over the past three years, our hospital encountered an extremely rare and unusual case. During the examination and diagnosis of a patient (5), we discovered a massive breast sarcoma. The tumor, nearly as large as a basketball, presented an exceedingly rare clinical scenario. This unprecedented size posed formidable diagnostic and treatment challenges, yet it also offered invaluable insights for medical research. We have meticulously documented and analyzed this case, hoping to contribute to the medical community through our detailed report. Present our unique clinical experience to peers. We aim to discuss and study the pathogenesis, diagnosis, and treatment strategies of this rare case to provide a reference for the diagnosis and treatment of similar cases in the future.

## Case description

A 43-year-old female was admitted in August 2021 due to a mass in the left breast. She initially noticed a small, painless mass (approximately 2.0 × 1.5 cm) in the left breast in February 2021, but ignored it without further evaluation. The mass gradually enlarged with accelerated growth, reaching 10 × 8.0 cm by August 2021. On presentation, the mass was hard, ill-defined, and non-tender. The patient denied fever, chest tightness, headache, dizziness, nausea, vomiting, or abdominal pain. Surgical treatment was recommended, but the patient refused and opted for self-treatment.

Her past medical history was unremarkable: no chronic diseases (such as hypertension, diabetes mellitus, coronary heart disease); no history of chest trauma or radiotherapy; no smoking or alcohol consumption; and no family history of genetic disorders or malignancies. No special treatment was administered outside the hospital. Physical examination showed no palpable lymphadenopathy in the bilateral axillae, supraclavicular regions, or neck; cardiopulmonary and abdominal examinations were unremarkable.

**Figure 1:**
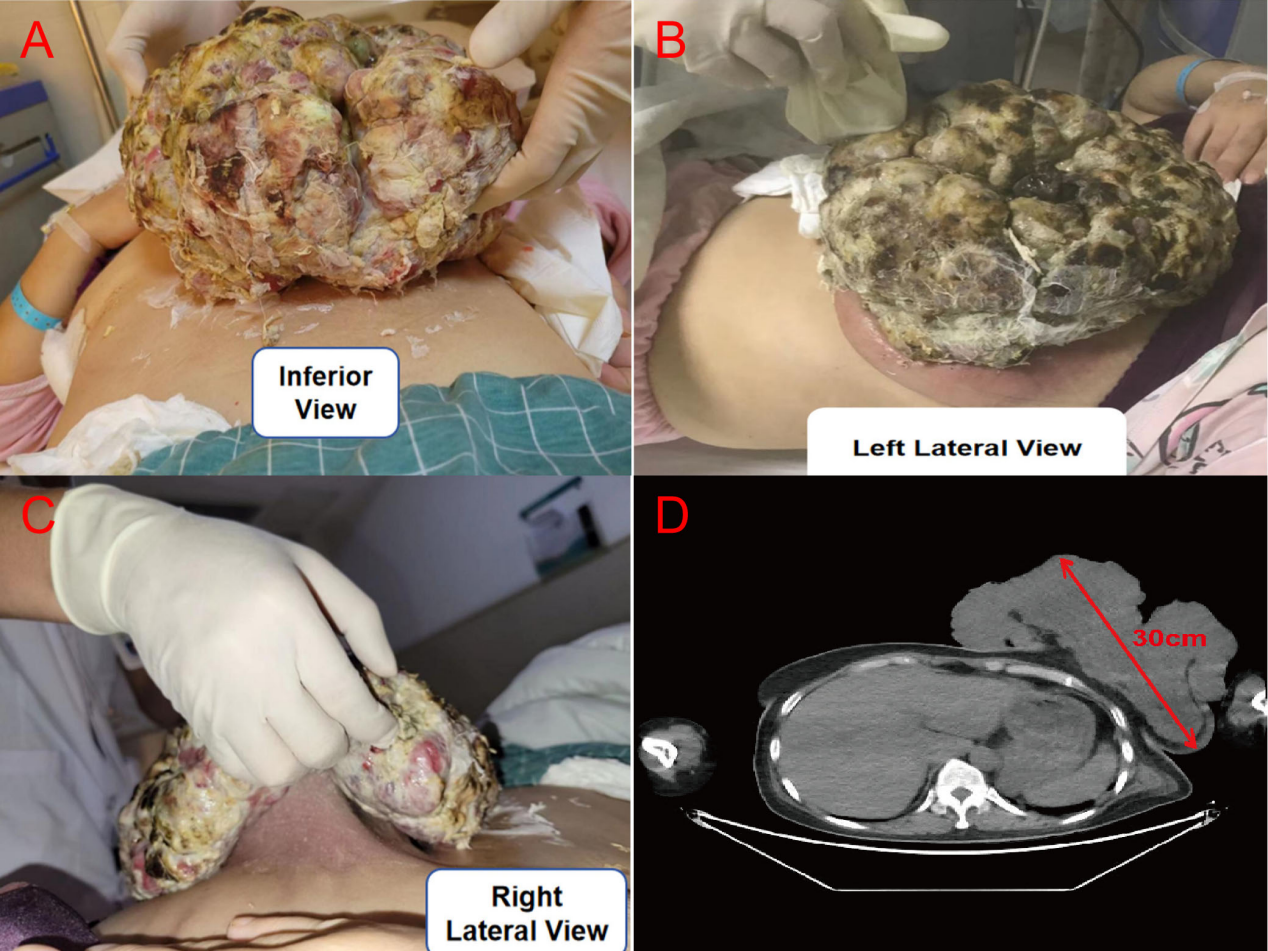
Clinical presentation and imaging of the giant pleomorphic undifferentiated sarcoma of the breast at admission. **(A)** Inferior view **(B)** Left lateral view **(C)** Right lateral view **(D)** Axial contrast-enhanced CT.

Within two months before admission, the left breast mass rapidly enlarged to 30 × 20 cm with ulceration. The ulcerated lesion showed a cauliflower-like appearance, accompanied by surrounding liquefactive necrosis, purulent discharge, and a foul odor (**Figures 1A–C**). Since the onset of the disease, the patient suffered from poor appetite and sleep quality. At admission, the giant tumor caused compression, leading to insufficient nutritional intake, systemic metabolic disturbances, and inflammatory responses. Laboratory tests revealed severe anemia, leukocytosis, and hypoalbuminemia: platelet count (PLT) 516 × 10^9^/L; leukocyte count (WBC) 21.10 × 10^9^/L; erythrocyte count (RBC) 2.14 × 10¹²/L; hemoglobin (Hb) 62 g/L; neutrophil count 18.70 × 10^9^/L; total protein (TP) 39.5 g/L; and albumin (ALB) 19.0 g/L. Tumor markers (female panel) showed elevated levels of carbohydrate antigen 125 (CA-125, 38.241 U/ml) and neutrophil gelatinase-associated lipocalin (250.59 ng/ml). Imaging revealed a giant mass in the left breast (**Figure 1D**), with no other significant abnormalities (6).

Supportive treatment was initiated immediately, including gastroprotective agents, blood transfusion, iron supplementation (ferrous succinate), hematopoietic growth factors, albumin infusion, and anti-inflammatory therapy. Subsequently, the patient underwent core needle biopsy of bilateral breast masses and fine-needle aspiration biopsy of the left axillary lymph node.

Puncture pathology results (7), pathology + immunohistochemistry: high-grade intraductal carcinoma was observed in the right breast puncture tissue; malignant tumor in the left breast, considered as sarcoma; fibro-adipose tissue in the left axilla, with no malignant lesion observed. Considering the patient’s poor physical condition, she continued to receive medication for blood replenishment therapy, blood transfusion, anti-inflammation, and infusion of nutrients. However, on October 7, 2021, the relevant indices were rechecked, and ultrasensitive C-reactive protein was 65.55 mg/L, total protein was 39.2 g/L, albumin was 19.7 g/L, and blood cell analysis revealed platelets at 502 × 10^9/L, leukocytes at 21.26 × 10^9/L, erythrocytes at 2.21 × 10^12/L, hemoglobin at 63 g/L, and neutrophil count at 18.90 × 10^9/L, procalcitonin at 0.161 ng/ml.

A multidisciplinary consultation was conducted with relevant departments. Combined with the opinions of each consultation department, the patient’s condition was complex, involving breast malignant tumor, hypoproteinemia, anemia, and possible renal disease. After a multidisciplinary consultation, a comprehensive treatment plan focusing on surgery was devised, coupled with anti-inflammatory, anticoagulation, and nutritional measures.

**Figure 2:**
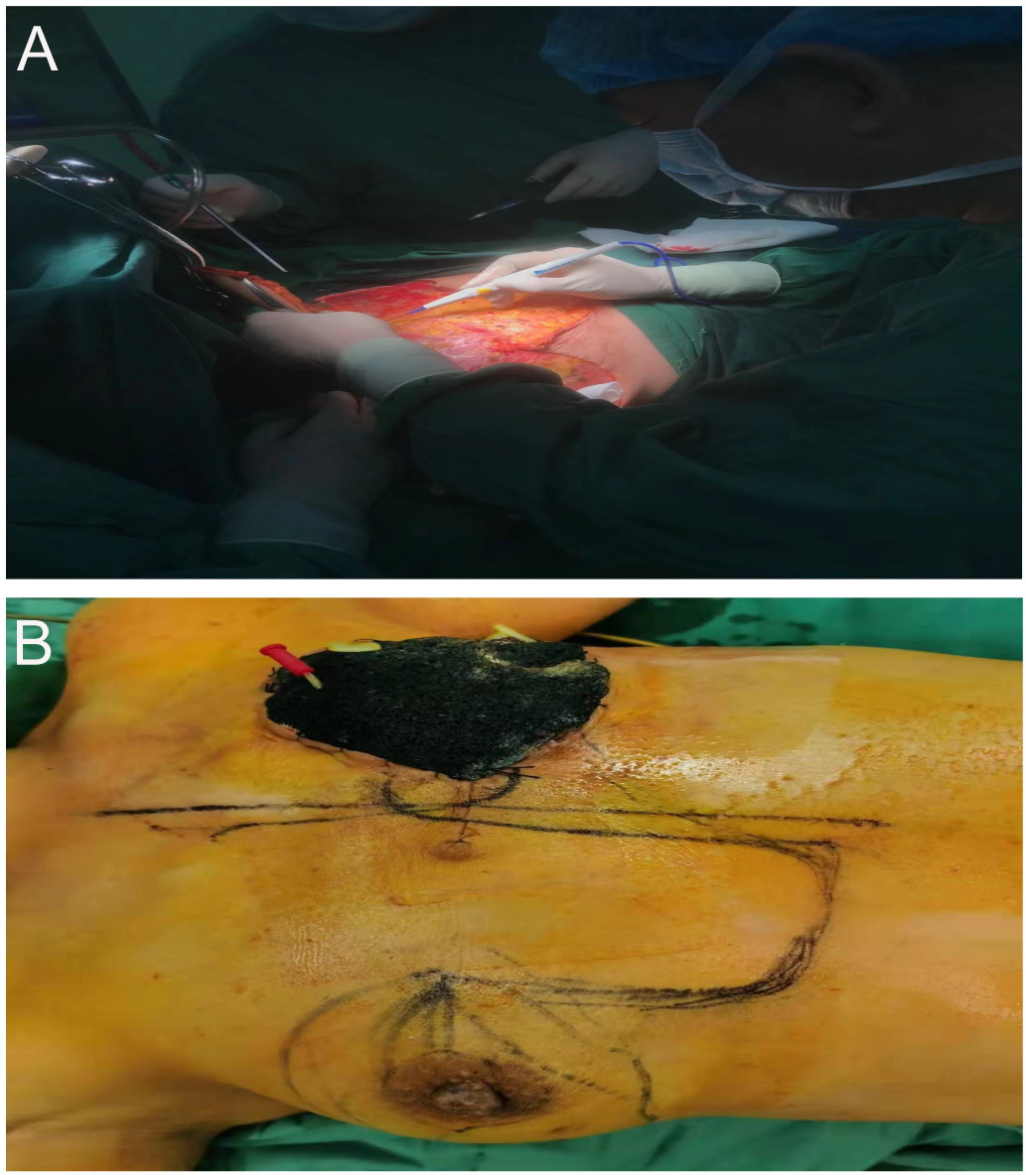
Surgical treatment of a giant sarcoma of the left breast. Image **(A)** details the surgical steps involved in the tumor resection; **(B)** VSD in negative pressure after left mastectomy.

The patient underwent left mastectomy, modified radical mastectomy, and vacuum-assisted closure (VSD) drainage. Intraoperative points of attention included: 1. Ensuring negative margins to prevent postoperative recurrence and metastasis; 2. The huge sarcoma, nearly 30 cm in size, was too large and heavy, requiring careful management of pus and blood around the mass to avoid infection of the trauma cavity; 3. The sarcoma had a rich blood supply, leading to significant intraoperative bleeding, which seriously affected the surgical field; 4. Active preparation of blood was necessary to manage intraoperative blood loss and avoid hemorrhagic shock; 5. Due to the inability to completely suture the incision, skin defects were managed with VSD negative pressure drainage; 6. Considering the patient’s physical condition, primary surgery for contralateral high-grade intraductal carcinoma was deemed too burdensome, and a second surgical treatment was recommended (**Figure 2**).

Postoperatively, the patient’s tumor load was significantly reduced, and her anemia and low albumin levels were quickly corrected. Antibiotics were continued to prevent incision infection. When the patient’s white blood cells, hemoglobin, albumin, and other indicators reached normal levels, a right mastectomy with ipsilateral axillary anterior sentinel lymph node biopsy and flap transplantation was performed on October 28, 2021. Before the operation, Director Li Xiangqi carefully designed the position and range of the transfer flap, ensuring it could fully cover the surgical defect and considering the survival rate of the transfer flap. Postoperatively, the patient’s vital signs and the transfer flap’s color, temperature, elasticity, and blood reflux were closely monitored, and effective psychological counseling was provided. The flap had good blood supply and remodeled its shape, and the patient and her family were very satisfied with the surgical effect and medical process. The patient regained confidence in life and started a new life.

**Figure 3:**
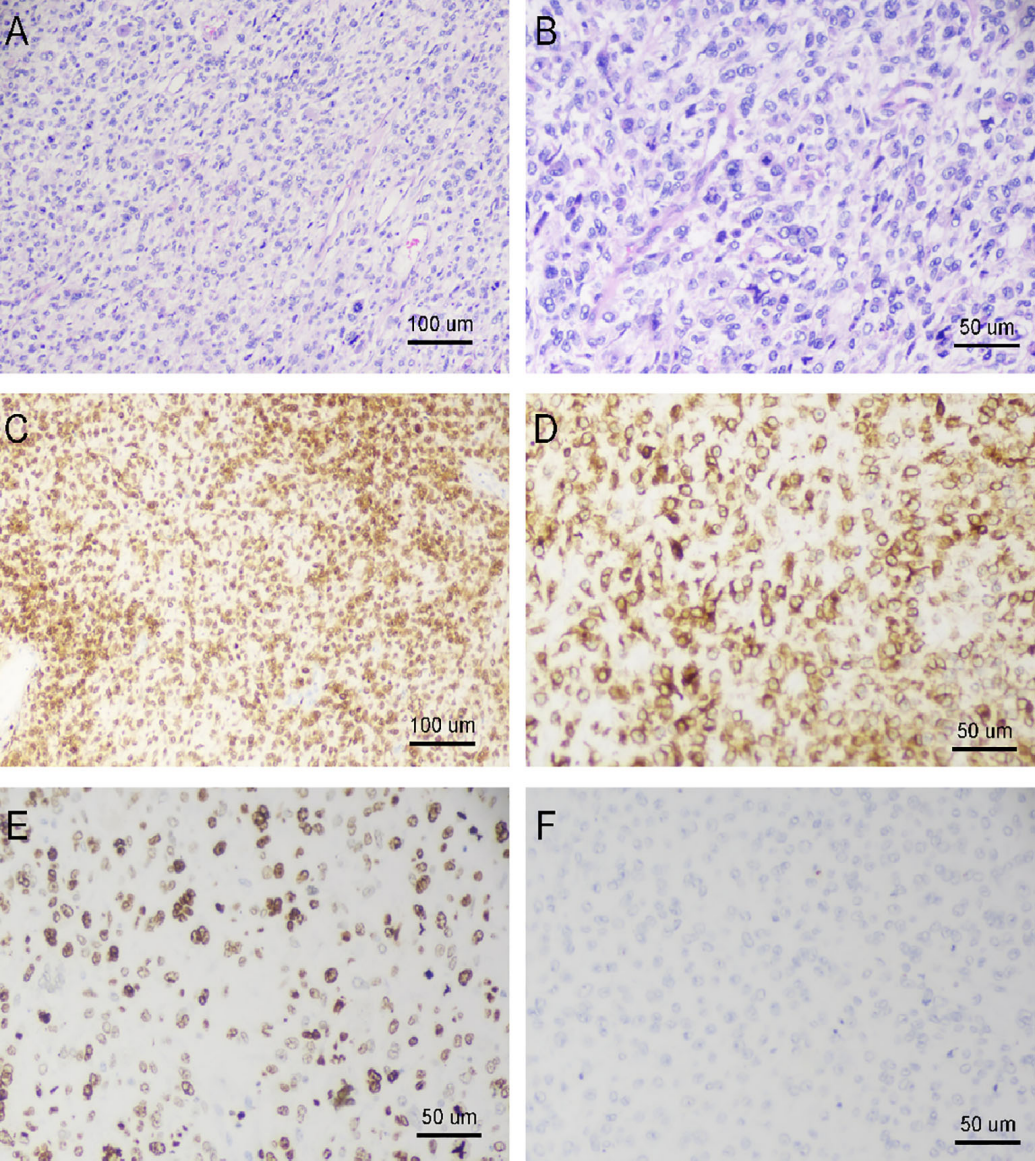
Histopathological Analysis of Breast Cancer Tissue Specimens. **(A, B)** Hematoxylin and -eosin (HE) staining of a breast tumor. A is a low magnification view and B is a high magnification view. **(C)** The case was an undifferentiated sarcoma of the breast, but was diffusely and consistently positive for P16 (10× objective). **(D)** Positive for vimentin. Positive staining for this waveform protein is suggestive of a tumor of mesenchymal origin and excludes the possibility of carcinoma (20× objective). **(E)** Images showing a high percentage of Ki-67 positive cells, reflecting the highly proliferative, malignant and invasive nature of the tumor. **(F)** Negative Expression of CK5/6. (20× objective).

Postoperative Pathology: left breast tumor and surrounding tissues (**Figure 3**) microscopic examination revealed a hypercellular mass with marked nuclear pleomorphism, visible multinucleated tumor giant cells, and increased mitotic figures.

Areas of cystic degeneration and large areas of necrosis were observed. Combined with immunohistochemical staining results, the findings were consistent with high-grade sarcoma, considered pleomorphic undifferentiated sarcoma (UPS). The tumor size was approximately 28 × 21 × 11 cm, adjacent to the base cut (<1 mm); self-examination revealed tumor tissue involvement and breakthrough of the skin surface. Axillary lymph nodes showed no tumor involvement (0/20). Immunohistochemistry: 202108550-A20: VIM (+), Ki-67 (50%+), B-Catenin (membrane+), BCL2(+), P63(+), P16(+), EMA (–), CDK4(-), D2-40 (partially+), CK5/6(-), STAT6(-).

Right breast radical specimen, The mass was entirely consistent with high-grade ductal carcinoma *in situ* of the breast, measuring approximately 3 × 1.5 × 1.5 cm. Autopsy of the fundoplication, skin, and nipple revealed no cancer involvement. Immunohistochemistry: 202108818-A: Ki-67 (15%+), HER-2 (2+), PR (<1%+), ER (>90%+), CK5/6 (myoepithelial+). Breast sarcoma was staged according to the soft tissue sarcoma staging criteria of the American Joint Committee on Cancer (AJCC), rather than the AJCC staging system for breast epithelial carcinoma (8). Based on the new TNM staging criteria for soft tissue sarcomas in the 8th edition of the AJCC (9), the patient was classified as stage IIIB (T4N0M0).

Postoperatively, the patient received radiotherapy to the left chest wall according to guidelines (10–12), including the surgical scar and the entire chest wall area to ensure coverage of possible residual cancer cells. The radiotherapy dose was 50 Gy in 25 fractions of 2 Gy, administered once daily, five days a week for 5-6 weeks. Given the patient’s high-grade ductal carcinoma *in situ* of the right breast with ER (>90%+) and PR (<1%+), and her premenopausal status, she was also prescribed Tamoxifen for endocrine therapy, 20 mg orally daily for 5-10 years. The patient was retested every three months, with no specific abnormalities or progression detected. A simplified clinical treatment timeline for the patient is presented in **Table 1**.

**Table 1:** Simplified clinical management timeline of the patient.

Date	Diagnostic/ intervention event	Key findings/outcomes
February 2021	Initial symptom onset	Painless left breast mass (2.0×1.5 cm); no medical consultation.
August 2021	Admission and preoperative evaluation	Left breast mass (30×20 cm) with ulceration and necrosis; abnormal lab indices (severe anemia, leukocytosis, elevated CA-125); CT confirmed giant mass; biopsy showed right breast high-grade intraductal carcinoma, suspected left breast sarcoma; nutritional support and anti-inflammation initiated.
October 7, 2021	Multidisciplinary consultation	Persistent abnormal inflammatory and nutritional indices; multidisciplinary evaluation completed.
October 2021	First-stage surgery (left mastectomy)	Left mastectomy and VSD drainage; negative margins, controlled bleeding; reduced tumor burden, corrected anemia and hypoalbuminemia postoperatively.
October 28, 2021	Second-stage surgery (right mastectomy with flap reconstruction)	Right mastectomy and sentinel lymph node biopsy and flap transplantation; postoperative psychological counseling.
Post second-stage surgery	Final pathological diagnosis	Left breast: Undifferentiated Pleomorphic Sarcoma (28×21×11 cm, skin invasion, negative lymph nodes); Right breast: High-grade ductal carcinoma *in situ* (3×1.5×1.5 cm, ER strongly positive).
Immediate postoperative period	Initiation of adjuvant therapy	Left chest wall radiotherapy (50 Gy total); Tamoxifen endocrine therapy (20 mg/d, planned 5-10 years).
2-5 years post-treatment	Regular follow-up	No tumor recurrence/metastasis; satisfactory flap survival and breast remodeling; improved quality of life, stable vital signs.

## Discussion

Giant sarcoma of the breast accompanied by anemia may indeed be associated with tumor-associated cachexia (13). Cachexia is a complex metabolic syndrome commonly seen in patients with advanced cancer, manifested by symptoms such as weight loss, muscle wasting, fatigue, and anemia. Advanced malignant disease is often accompanied by systemic metabolic disorders (14), In such cases, metabolic disturbances may persist despite supportive measures, and blood supplementation alone is often ineffective.

Several factors may contribute to hypoproteinemia in patients with giant breast sarcoma (15), including tumor consumption, hypoxia, impaired liver function, malnutrition, and inflammatory response. The key to treatment lies in: surgical resection of the tumor, direct removal of the cause of the disease, improvement of the metabolic state, combined with anti-inflammatory treatment, chemotherapy or radiotherapy, to improve the patient’s condition comprehensively.

Elevated inflammatory markers (e.g., C-reactive protein (CRP), elevated white blood cell count, etc.) accompanying giant breast sarcoma may be related to tumor necrosis and its triggering of a systemic inflammatory response (16). Benefits of tumor resection: elimination of necrotic tissue and sources of inflammation, reduction of the release of inflammatory factors, and elimination of systemic inflammation.

P16 protein is an important cell cycle regulatory protein that regulates cell cycle progression by inhibiting CDK4/6 activity (17). Notably, the combination of positive P16 expression with negative MDM2 and CDK4 expression in this case offers crucial evidence for excluding dedifferentiated liposarcoma. While dedifferentiated liposarcoma usually exhibits overexpression of MDM2 and CDK4, both markers were negative in this case, further supporting the diagnosis of undifferentiated pleomorphic sarcoma. This finding suggests that the combined use of markers such as P16, MDM2, and CDK4 in the diagnosis of undifferentiated sarcoma can help to more accurately identify the tumor type, thus providing a more reliable pathological basis for clinical treatment.

Although several cases of undifferentiated pleomorphic sarcoma of the breast have been reported in the literature (18, 19), cases as massive as the present one remain extremely rare. Meanwhile, although focal P63 expression has been documented in a small number of studies on undifferentiated pleomorphic sarcoma (20), its expression pattern differs significantly from the characteristic diffuse and strong positivity seen in metaplastic carcinoma, which allows for reliable differential diagnosis. In conclusion, the absence of epithelial morphological features, negative epithelial markers, and partial P63 positivity definitively exclude metaplastic carcinoma and support the diagnosis of undifferentiated pleomorphic sarcoma (UPS).

## Conclusions

Undifferentiated sarcomas are a group of highly aggressive and heterogeneous malignant tumors and distinguishing them from metaplastic carcinoma is critically important given the differences in their respective treatment strategies (21). Although chemotherapy is a common treatment, the results are often unsatisfactory. This case highlights the importance of a multidisciplinary approach, incorporating surgery, radiotherapy, and systemic therapies, to optimize patient outcomes. The association between tumor necrosis, systemic inflammatory response, and hypoalbuminemia underscores the complex interplay between tumor biology and host response. Further research is needed to elucidate the molecular mechanisms driving this disease and to identify targeted therapies that can improve survival and quality of life for patients with this rare and challenging condition.

## Patient perspective

In February 2021, I presented with a painless mass in the left breast; due to fear of surgery, I did not receive timely intervention, and the tumor subsequently enlarged rapidly with ulceration and malodor. I was diagnosed with giant undifferentiated pleomorphic sarcoma of the left breast and high-grade ductal carcinoma *in situ* of the right breast. I underwent bilateral mastectomy, with defect drainage following left mastectomy and flap reconstruction after right mastectomy, followed by radiotherapy and endocrine therapy. With the management and support of the multidisciplinary team, I completed the entire treatment course successfully and remain alive and in stable condition. This experience serves as a reminder for patients with rare breast tumors to pursue early diagnosis and treatment, receive standardized medical care, and avoid delay in seeking medical attention.

## Data Availability

The raw data supporting the conclusions of this article will be made available by the authors, without undue reservation.
